# The effectiveness of anti-vaping health communication campaigns among high school and college students in the U.S.

**DOI:** 10.3389/fpubh.2025.1676181

**Published:** 2026-01-05

**Authors:** Sarah R. Skran

**Affiliations:** 1 Criminal Justice, Monmouth University, West Long Branch, NJ, United States; 2 Independent Researcher, Marlboro, NJ, United States

**Keywords:** anti-vaping, prevention campaigns, health communication, PSAS, warning labels, message themes, adolescents, young adults

## Abstract

**Introduction:**

This policy review looks at the exact message themes used in anti-vaping health communication campaigns, PSAs, and warning labels to determine how they can be improved to decrease vaping initiation and encourage cessation among adolescents and young adults.

**Literature review:**

Two meta-analyses, one on anti-vaping campaigns and another on vaping advertisements are reviewed in addition to one retrospective on tobacco warning labels. Overall, messages that reveal specific health harms and increase perceived risk are most likely to decrease vaping tendency.

**Methods:**

A total of 16 studies are analyzed assessing the viewpoints of 21,427 people regarding how they feel about terminology used in several types of anti-vaping messages.

**Actionable recommendations:**

Multiple recommendations are made regarding how to improve the efficacy of current anti-vaping campaigns. Among several other factors, messages that denote the health consequences of vaping and chemicals contained in vapor products are the most effective. Meanwhile, messages that warn that nicotine is addictive are deemed the least effective.

**Discussion:**

Implications of these findings for policymakers include the need for a new FDA warning label that includes specific health implications of vape and nicotine use; Structural changes to restrict youth and young adult access to vape products; as well as guidance regarding how to use social media to disseminate information on the dangers of vaping.

## Introduction

The topic of this review is anti-vaping campaigns and their effects on vaping rates among adolescents and young adults. Namely, whether or not they encourage cessation and/or decrease vaping initiation. Presently, numerous anti-vaping campaigns and warnings have been adopted by federal, local and state agencies with the most prominent being the FDA's *Real Cost* campaign and the Truth Initiative's *Truth* campaign. The FDA, responsible for regulating all tobacco products as of 2009, has required a label on vapor products warning users that nicotine is addictive since 2018 (1). Additionally, as part of their Youth Tobacco Prevention Campaign they launched their anti-vaping PSA *The Real Cost* in 2019. *The Real Cost* geared toward a younger audience of 12–17 year olds (2), highlights the negative health outcomes of e-cig use through TV ads, online videos, social media, and streaming platforms (3, 4). Similarly, the *Truth Initiative* a non-profit organization mirrors the FDA with its National Anti-Tobacco Campaign. However, their PSAs are slightly different in that they gear them toward an older audience of 15–24 year olds (2), with a focus on exposing industry practices and presenting young people with facts about vaping (5).

Beyond these, there are a number of state and local organizations that have launched similar prevention campaigns including school-based programs. One of these is the 2015 *Still Blowing Smoke* campaign and 2018 *Flavors Hook Kids* campaign developed by the California Department of Public Health (6). In Mississippi, *Generation Free* was launched for high school and university students that uses scientific information about the health effects of e-cigs, the chemical composition of e-cig aerosols, and relationship between e-cigs and cigarettes to reduce interest in vaping (3). Another school-based program is *Project EX* which teaches adolescents about the dangers of vaping as well as how to deal with peer pressure (5). Finally, *Behind the Haze* by *Rescue Agency*, launched in 2019 and implemented in 12 states as of 2022, consists of a commercial with corresponding radio, video, digital and social media content. The campaign provides reasons for non-users to avoid initiation and for vapers to quit by relaying basic knowledge about vaping, establishing risk, and creating urgency and relevance (7).

### Trends in vape use

Prevention of vaping initiation and cessation is of extreme importance as rates of nicotine use and subsequent health consequences among youth and young adults have risen exponentially in recent years. Due to successful public health messaging regarding smoking, cigarette use decreased from almost half the population (42%) in 1965 [(8), p. 735] to just 11% in 2022 [(9), p. 635]. Unfortunately, one drawback of these initial campaigns is they largely ignored the consequences of using other tobacco products including e-cigarettes. Initially, e-cigarettes were created as a novel way to help adult users of traditional cigarettes quit smoking. The goal was to improve outcomes for users who had tried other smoking cessation methods but failed to successfully quit smoking. Traditional cessation methods include Nicotine Replacement Therapy (NRT) such as nicotine gum, patches, inhalers, lozenges and medications like Zyban and Chantix (10). These tend to have very low success rates with only 10% [(10), p. 2] of users quitting smoking for more than 6 months. As such e-cigarettes have been adopted by many as an alternative means to attempt to quit smoking. Evidence supports this, finding that 1 year abstinence from smoking is about 10% higher among those who use e-cigarettes (18%) compared to those who use NRT (9.9%) [(10), p. 4]. The success of the former over the latter is likely due to the fact e-cigs are more satisfying to ex-smokers since they mimic the look and feel of smoking in addition to having more flavors and a higher nicotine content relative to other smoking cessation methods. The issue is that many non-smoking young adults and teens like these products for similar reasons, resulting in e-cigs being used for the opposite purpose for which they were created, namely to initiate nicotine use (10). Unfortunately, when e-cigarettes first hit the market, health communication campaigns had not quite caught up with the times and by the time they did the damage was significant.

Many young adults and youth starting using them despite never having smoked previously. In fact, in 2015, 32.5% of vape users were never smokers [(5), p. 57]. At the time there were few if any studies indicating e-cigs could cause severe health consequences among non-smokers and because they did not contain tobacco many individuals including youth thought they were harmless. In turn, there would be a 900% [(11), p. 1] increase in teen e-cig use going from only 1.5% in 2011 to 16% in 2015 [(8), p. 735]. 2014 would mark the official transition by teens from combustible tobacco cigarettes to e-cigarettes as they became the most commonly used tobacco product among high school aged youth (12). In the years that followed, with the release of JUUL in 2017, vaping would become increasingly popular among adolescents and young adults. 2019 represented the highest vaping rates ever recorded reaching 27.5% among high school students, the same percentage that smoked in 1991 [(12), p. e228]. This was a 1,800% increase since 2011 [(6), p. 1]. This resurgence of tobacco use raised significant concerns leading the US Surgeon General and FDA to describe vaping as an epidemic among youth. Since then, vaping rates among highschoolers have drastically decreased dropping from 19.6% in 2020 [(13), p. 1881] to just 10% in 2023 and 7.8% in 2024 [(14), p. 917]. However, among college students the percentages remain high with 18% of college students reporting current use of nicotine vapor products in 2023 [(15), p. 47]. The implication of this is that anti-vaping messages are more successful among younger audiences but are failing to resonate with older audiences. Another interpretation of this is that fewer highschoolers are initiating use while college students are continuing use that started in high school. If this is the case, this would imply that these campaigns are better at deterring initiation rather than promoting cessation. Regardless, current e-cig use trends do show a vast improvement. With that being said, they still remain concerning when compared to 1.7% [(14), p. 918] of highschoolers and 4% of college students [(15), p. 47] who smoke cigarettes suggesting a significant number of vape users still believe vaping is a safe alternative to smoking.

### Health impacts

E-cigarettes, otherwise known as vapes, contain some of the same chemicals as their combustible counterparts causing similar health issues. When heated, the vapor emitted by these products has been found to contain carcinogens like formaldehyde as well as metals like lead and arsenic. In the short term these and many other toxins found in vaping aerosols can lead to permanent lung damage, DNA damage (which is a known cause of cancer), as well as numerous respiratory and cardiovascular issues such as a weakened immune system, high blood pressure, stroke, and heart attack (16). More specifically vaping has been linked to pneumothorax typically referred to as “collapsed lung” in teens and young adults (17–19). As a result, numerous teens and young adults have been hospitalized requiring painful procedures and sometimes even surgery in which chest tubes are inserted into the lungs (18). There are numerous theories for why this happens, including lung injury (17), ruptured blisters (18), chemicals (18) and inhalation techniques (17). In some instances, Vitamin E acetate (18, 19), nicotine salts (17) and certain kinds of flavoring (17, 19) have been blamed specifically while in other scenarios it is less obvious what chemical or specific action was the culprit of the problem.

In a systematic review of vaping related cases of pneumothorax, a quarter of individuals reported cigarette smoking, and even more were cannabis users, making it difficult to isolate vaping as an independent risk factor. Furthermore, due to under-reporting of vaping there may be more cases of collapsed lungs in young people but because the person did not honestly divulge their vaping or smoking history, it is hard to say how likely vaping is to cause pneumothorax (18). What can be stated for certain is that those who vape frequently report having trouble breathing and chest pain (20) which are also common symptoms of a collapsed lung (17–19) indicating vaping causes respiratory issues (19). With that being said, although we know more today about the short-term physiological implications of vaping, it will be decades until any long-term effects will be fully understood. Perhaps even more concerning is the fact most teens lack this basic knowledge causing them to downplay the risks of using e-cigarettes and nicotine addiction in general (7). As such, it is essential to try to understand how to improve anti-vaping campaigns to prevent a surge of vaping related illness and potential death.

### Theoretical framework

Risk perception of vaping and subsequent behavior or vape use can be explained by the health belief model (HBM). This model was specifically created to encourage people to take actions to prevent illnesses that are often asymptomatic, or only result in symptoms years later. As such it is commonly used to encourage cessation of unhealthy behaviors such as smoking. HBM posits that susceptibility to a health problem or health consequences and the severity of the condition form a person's perception of the health threat. The higher the threat, the more likely they are to take action or change their behavior to reduce their chances of experiencing that health condition (21). In reference to the topic of this paper, changes in behavior might include not initiating vape use or ceasing the behavior if one has already started. Examples of things that may increase risk perception include exposure to health campaigns or witnessing others experiencing health consequences (21). Social media influencers enduring the negative effects of vaping would be an example of the latter. With that being said, peer influences can also have the opposite effect, encouraging the person to use in order to fit in. Self-efficacy or adherence to a cessation plan may also be made more difficult by physical addiction to nicotine and the perception that it is enjoyable or relieves stress. Furthermore, if a person does not believe they are likely to experience consequences or don't think the health issues are serious enough they are less likely to take preventative action (21). This therefore demonstrates the necessity for research on how to improve health communication campaigns in order to enact change in current vaping behavior.

### Scope/topic of this paper

This policy paper helps to improve current anti-vaping campaigns by clarifying what specific message themes are most likely to deter vaping. While mass media campaigns and warnings have been generally effective when it comes to traditional tobacco products, the same cannot be said about anti-vaping messages, which have only recently started to be studied regarding their efficacy. While some claim that anti-vaping public health campaigns are working others question if this is true. For instance, in could be argued that the *Truth* and *Real Cost* campaigns inadvertently promote vape use by mimicking the vaping advertisements they are trying to combat. They do this by depicting vape pens with bright colors and fruity flavors being used by young people (22). Some of their PSAs have also made exaggerated claims about the correlation between depression/anxiety and vaping (3, 4, 7). Meanwhile others completely miss the mark trying to mimic teenagers with teenage vernacular (2) or focusing on issues most teens discount or don't care much about like addiction (4) and environmental impact.

With regard to the studies themselves they often focus too much on health harms and addiction themes rarely accounting for other themes (23). They also fail to address the specific terminology used in these messages. In fact, most studies provide no recommendations for improvement. Instead, they simply state that the anti-vaping advertisements are effective even if many of them failed to change beliefs about the risks of vaping (4). Some studies are even funded by the FDA, resulting in minimal critiques if any of the FDA's anti-vaping policies or campaigns. For example, the current FDA warning on vape products focuses solely on nicotine being addictive, despite evidence which indicates packaging with an explanation of the health harms of vaping would be more impactful (23). Thus, this paper will attempt to fill in these gaps by examining these elements further and making specific recommendations for how to improve anti-vaping messaging.

For clarification, this paper focuses on the use of e-cigs by never smokers. The study of the effectiveness of e-cigs in smoking cessation is out-of-scope. Nothing in this paper regarding the harms of e-cig usage should be interpreted to mean that e-cigs should not play a vital role in smoking cessation under an appropriate regulatory system.

## Literature review

### Anti-vaping campaigns

For this literature review two meta-analyses and one historical retrospective on tobacco prevention messages are discussed. The most relevant of these is a meta-analysis on the efficacy of anti-vaping messages including videos, text statements, or warning labels and is the first of its kind. The researchers included a total of 6,622 people up to 25 years old in their sample frame with 7 studies focusing on young adults and 5 on adolescents. All 12 studies were conducted in the US between the years 2016 and 2021 and focused solely on vaping prevention rather than cessation. Three categories were deemed most relevant to vaping prevention messages by the researchers. These were message reactions, risk beliefs, and intentions. *Message reactions* refer to the impact a message had on feelings of anxiety or fear. The studies indicated no significant change in message reactions relative to those who weren't exposed to them. This was likely due to the fact that these messages lacked images of health consequences which are a key mechanism of persuasion. Moving on to *risk beliefs*, these refer to the risk perception an individual has about engaging in a particular behavior. This is important to test because risk beliefs go hand in hand with altering behavior. Finally, behavior is influenced by one's *intentions*. The results indicated that vaping prevention messages focused on the health effects and consequences of vaping increased the belief that vaping is risky. However, the decrease in vaping intentions was less significant, suggesting some may continue to engage in vaping behavior even if they are fully aware of the risks (24).

Regarding limitations, the kinds of anti-vaping messages noted in this meta-analysis lacked the specificity needed to make any actionable changes to current anti-vaping campaigns. For instance, it mentions that vaping prevention messages focused on the health consequences and addictive nature of vaping but did not explain what these consequences were or how they were presented. This essay hopes to expand upon the above literature by assessing the specific statements and terminology used in anti-vaping messages and campaigns to improve their efficacy. Furthermore, following the recommendations for future research made by the authors, this review will assess studies that exposed individuals to anti-vaping messages over time and in real world contexts such as social media.

### Vaping advertisements

The next meta-analysis is also related to vaping but in contrast to the above article, examined consumer response to vaping advertisements. This meta-analysis was far larger and more expansive, analyzing a total of 43 papers and 140 studies making 77,452 observations. The population of the final sample encompassed a wide range of individuals from several countries (US, UK, Canada, New Zealand, Germany, China, and Indonesia) as well as smoking and non-smoking adults and adolescents. Similarly to the previous meta-analysis, it was determined that *perceived risk* reduces the effect vaping advertisements have on vaping tendency. In other words, perceived risk can impact how successful a vaping advertisement is at convincing the viewer to vape or try their product. Thus, Yang and his colleagues (5) propose that educational programs should aim to change individuals perceived risk of vaping by warning consumers about the health risks associated with vaping while also ensuring the contrasting social motivations for vaping are addressed.

Other relevant findings revolved around contextual factors such as advertising platform, presentation mode, consumer age and consumer smoking status. To further explain, it was ascertained that adolescents and non-smokers were more vulnerable to vaping advertisements than their adult and smoking counterparts. This indicates that the target audience of anti-vaping campaigns should be those most influenced by vaping advertisements, namely adolescent non-smokers. In addition to this, social media and picture dominant cues were also deemed to be most impactful. This would suggest that social media should be used to spread anti-vaping messages and that images are more likely to be effective than words [(5), Table 6].

This policy review will attempt to confirm these theories by assessing anti-vaping educational campaigns geared toward teens and young adults. Specifically, those that include pictures of the health impacts of vaping, utilize social media to spread their messages, and address issues like peer pressure. In all, the correlation between risk beliefs and intentions will be examined, in particular what kinds of beliefs are most likely to decrease vaping behavior.

### Cigarette warning labels

The last article that will be noted in this literature review references multiple studies discussing the effectiveness of tobacco product warning labels thus making it pertinent to discuss here. The article, published in the *Chapman Law Review* and written by Blum (1) of the Loyola University Chicago School of Law, gives a historical perspective on tobacco control. The first study Blum mentions was presented in a report by the Federal Trade Commission (FTC) to Congress in 1967, just 2 years after the *Cigarette Labeling and Advertising Act* was enacted in 1965. The act required a warning be placed on cigarette packages stating they were hazardous to health. However, based on survey data collected from public health professionals and consumers the warning did not have any effect on cigarette consumption; in fact, product sales increased the first 2 months after the warning label appeared. According to respondents of the survey, the label language was insufficient because it lacked specificity. Thus, the commission recommended warning messages identify specific health harms like cancer as well as state the nicotine content of the product. Unfortunately, due to push back from the tobacco industry the only change that was made in response to the report occurred in 1970, with the passing of the *Public Health Smoking Act*, adding to the warning label that the source of the cautionary statement was from the Surgeon General (1).

As one would expect, according to another report made by the FTC to Congress in 1981, the warnings were not effective enough to change public knowledge and people's attitudes toward smoking, leading to the *Comprehensive Smoking Education Act of 1984*. This act would finally change the warning label on cigarettes to include specific health implications such as the impact of smoking on pregnant women and the toxins found in cigarette smoke. Almost 30 years later in 2009 the FDA per the *Family Prevention and Tobacco Control Act*, would recommend complimentary pictures be added to the labels to increase the effectiveness of the warning messages. The pictures chosen stemmed from an analysis of 36 graphic images drawn from consumer research on health communications that indicated images that evoked an emotional response were more likely to grab consumers attention. Based on an internet survey of 18,000 people, images of empowerment, diseased organs, people and death, were found to be the most likely to increase the desire to refrain from smoking and expand knowledge about the risks of smoking and secondhand smoke (1).

Thus, based on these findings, Blum (1) recommends similar measures be taken to change the current label on vapor products to include both text and images that explicitly define the dangers of vaping. Stemming from Blum's findings, this policy review offers specific recommendations as to what these labels should say and how they should be presented. Namely, messages which result in the highest levels of fear and anxiety should be prioritized (1). In essence, while there is a general consensus that the kind of messages that are most impactful on vaping rates are those that allude to specific health effects, the aim of this review will be to explain in further detail how they can be shared in the most effective manner.

## Methodology

### Selection criteria for study inclusion

All studies chosen for analysis as part of this review had to research the effectiveness of vaping prevention messages. These messages could refer to video PSAs such as the *Truth* or *Real Cost* social media campaigns, posters found on school campuses, or warning labels. In essence anything that communicated an anti-vaping message whether it be via words, audio or visual means counted as a vaping prevention message. Regarding specificity, the studies had to describe the kind of messages discussed in them. To be more specific, what was said in the anti-vaping message or what elements made it effective or ineffective had to be noted. Additionally, this review focuses on cessation as well as initiation. Thus, a combination of vapers and non-vapers are surveyed, including some smokers. This was decided since previous use of nicotine products may impact the influence vaping campaigns have on participants. Next, though not technically a criterion, it turned out all studies were conducted in the U.S., with seven using nationally representative samples. Of the studies that were not nationally representative, participants included those from Hawaii, Indiana, Iowa, Kansas, Kentucky, Nevada, Oklahoma, North and South Carolina, Vermont, and Virginia. In turn, this made the results of this analysis characteristic of the nation as a whole, with most statistics presented in survey results matching those in national survey data.

Furthermore, no limit was placed on when study data was collected. As a result, some study data was collected more than 10 years ago covering products that are no longer on the market. Despite this, older studies remain eligible for this review since they produce actionable insight into the kinds of elements that make public health messaging effective. Thus, data collection for the chosen studies took place between the years 2014 and 2021, representing the vast history of vapor products ranging from cig-alikes (blu, MarkTen and NJoy) to bulky refillable devices, to sleek pod-based systems (JUUL), and last but not least, to disposable vapes with thousands of puffs worth of e-liquid. Finally, based on the target audience of anti-vaping campaigns this analysis primarily includes studies conducted on young adults and adolescents. Except for one study that did not limit the age of adults that could participate, the adult studies focused on those who were in their 20s. Eight studies were conducted on adolescents including both middle and high school students, four studies on adults including parents and four on both adolescents and adults. In all, this totaled 16 studies and 21,427 people.

### Search process

To start, all 12 studies cited in the first meta-analysis on anti-vaping campaigns (24) were reviewed. After some were excluded due to inaccessibility or because they were not specific enough, only 6 remained. The other 10 studies included in this review were discovered via Google Scholar. Search terms included the “impact” and “effectiveness” of “anti-vaping” “PSAs”, “warning labels”, and “prevention campaigns”. Studies chosen were based on what was written in the abstract. In some cases, more reading was required in order to determine if a study was eligible for inclusion. Once again, some studies did not fulfill the search criteria for study inclusion. Additionally, some studies were not included because they were behind a paywall. As such, all studies chosen were either free or could be accessed using university credentials. This may have resulted in some selection bias. Therefore, open-access prioritization is suggested for future reviews. The electronic databases where the studies were available for download included JSTOR, EBSCOhost, PubMed, ScienceDirect, ProQuest, ElSEVIER Scopus, Routledge, APAPsycNet and Sage Publications. The final set of 16 studies chosen and a summary of their findings can be found in Supplementary Table 1. Furthermore, a PRISMA flow diagram depicting the selection process at each stage can be found in Supplementary Figure 1.

## Actionable recommendations based on key findings

### Health effects

Messages that communicate the health effects of vaping and chemical constituents of vapes are deemed the most effective. However, there are some guidelines that need to be followed when discussing these themes. First in reference to health effects, it is not wise to state that the long-term health effects are unknown (2, 11) as this may serve as a justification for use (3) by increasing ambiguity perceptions which leads to a process that increases intentions to vape. Therefore, instead of focusing on what little is known about the health effects of vaping the focus should be placed on what is known (i.e. the short-term effects). Since most teens and young adults discount long term health effects anyway due to temporal discounting (25) this is probably advantageous. Furthermore, the short-term effects of vaping are proving to be far more severe than most could have imagined, making them more likely to dissuade people from vaping (17–19). With that said, those who are unaware of or do not believe in the short-term consequences of vaping are more likely to vape (26), demonstrating the need to mention them in anti-vaping campaigns.

Generally, messages should avoid vague statements about health risks such as having difficulty breathing (20) and instead allude to specific health conditions. In other words, anti-vaping campaigns should describe exactly how vaping damages the lungs or other parts of the body (7). For example, vaping can cause blisters of air to form in the lungs (18) as well as cell and tissue damage (17) which can cause one's lungs to collapse (17–19). To help demonstrate this, visuals ((19), Images 1 and 2) should be used as these are perceived as evidence of the damage vaping can do to the body (16, 23). Graphics may also help those who do not recognize the name of a disease (or chemical) to better comprehend the message presented (7). Images depicting internal harms like diseased organs or persons experiencing harms are perceived as the most discouraging (16). In reference to the latter, showing a worried family at the bedside of a hospitalized vaper is considered particularly effective because kids do not want to cause their family distress (7).

### Relative harm statements

Relative harm statements compare the health effects of one product to a similar product. They are usually placed on vapes and e-cigarettes as these contain nicotine but not tobacco. The intention is to warn the user that even though the product isn't entirely safe as it still contains nicotine, it is much safer than its tobacco counterpart. This is shown to increase the use of e- cigarettes for harm reduction purposes by smokers. The concern is that it may also encourage vaping initiation among non-smokers. This is based on studies that find messages that contain relative harm statements in addition to other statements describing the risks of vaping, reduce recall of the risk related part of the message therefore increasing vaping appeal (27). This comparative statement also tends to reduce message comprehension (28), making the message sound less believable and credible (23). Therefore, it seems while well intentioned this is not a helpful addition to anti-vaping messages.

### Chemical content

According to a 2023 study only 70% of chemical messages in existing anti-vape campaigns named the specific chemical and only 41% mentioned where else the chemical could be found [(7), p. 415]. This is concerning due to the fact messages with this theme are only effective when they are specific. In other words, it is not enough to simply state that a vape has chemicals in it. The PSA must explain what harms the chemical causes and why it is harmful. This is key to overcoming the general apathy that people have toward chemical exposure (7). For example, formaldehyde causes DNA damage which leads to cancer (16). Furthermore, for chemicals that are unknown, it may be useful to explain where else the chemical can be found to demonstrate its toxicity (7). For instance, arsenic is found in bug spray (12), pulegone is found in insecticide (7), and acetone is found in nail polish remover (6). Other chemicals contained in e-cigarette vapor though not specifically referred to are contained in sewage and paint (8). With that said, if the chemical is contained in a household item that is traditionally viewed as safe and non-toxic like glycerin which is the main ingredient in soap, this technique is not recommended (7). In cases like these it may be more helpful to simply explain how the chemical can be toxic to the body. For instance, in the case of glycerin, when heated the substance turns into acrolein which is known to cause permanent lung damage. Finally, in terms of pictorials, images of the internal harms these chemicals and/or metals cause are more likely to serve as a deterrent than images of the hazards themselves (16, 23).

### Statistics and facts

Providing statistical and other scientific fact-based information is essential when trying to convince teens of the dangers of e-cigarettes (8). This is especially true in the modern world in which there is a plethora of fake news, and it is difficult to distinguish between fact and fiction. Thus, all claims made in PSAs must be further substantiated by a specific study or set of studies otherwise they are unlikely to be taken seriously. In turn, this can be an effective means of sharing health information with teens as it empowers them to make their own decisions as opposed to having an authority figure tell them what to do. With that said, some individuals complain that even with valid sources, certain statements are hard to believe. For instance, evidence suggests that youth overestimate how much their peers vape (22). For example, one anti-vaping message based on data that found only about a quarter of college students vape, stated that most college students were choosing not to use e-cigarettes. This significantly conflicted with the participants' perceptions of pervasive use among their peers and therefore was not convincing enough, even if technically true (3). This suggests that study results while sometimes helpful may not be sufficient to significantly change risk beliefs about vaping among teens and young adults (23). As such, they should not be used as the sole means to convince youth of the dangers of vaping.

### Nicotine addiction

Nicotine addiction is one of the most common themes of vaping prevention messages. Ironically, there is evidence that they are also some of the least effective as the majority of teens and even adults do not have an inherently negative perception of nicotine. This can be attributed to the inability of tobacco prevention campaigns to illustrate the danger of nicotine use. To explain, most messages that address nicotine addiction focus on the withdrawal symptoms like irritability, mood swings, headaches (6) and the effect of nicotine on the brain (22). More serious health consequences of nicotine use such as increasing the heart rate and blood pressure, which can lead to heart disease and heart attack (28) as well as infertility [(29), Table 1] are ignored. The problem with this is that withdrawal symptoms are not only not significant enough to deter use among non-users, but they may also inadvertently encourage users to continue to vape in order to avoid experiencing them. Therefore, to prevent initiation of the substance it may be preferable to alternatively explain in these messages that once one becomes a regular user of nicotine, the buzz that is often cited as a benefit of using nicotine (3) will dissipate. Meanwhile to encourage cessation, using the word “addiction” should be avoided as these messages are often viewed as accusatory, eliciting a defensive reaction among vape users. This response is the result of denial and a refusal to believe one is addicted, which is common among drug users. Thus, instead of using the word “addicted” in the message, researchers recommend messages make more indirect statements about the signs of dependence. These indicators should reference the transition from social to regular use which may involve purchasing the device for oneself or using the device on a daily basis [(3), Figure 2].

### Financial cost

One of the primary consequences of nicotine addiction is the financial cost. But unlike withdrawal symptoms, this aspect of addiction is more likely to increase the perception that cessation will yield benefits due to its positive connotation (3). Additionally, as opposed to health effects, it cannot be discarded as lacking evidence (6). Thus, the financial impact of nicotine addiction is a promising message theme according to researchers. Regarding what should be said specifically, denoting the amount of money vaping costs as opposed to simply stating that it is costly is preferred (3). To assist with this, interactive messaging strategies like cost calculators or equivalency data could be used (8). In reference to the visuals in the advertisement, images should include examples of alternative purchases that could be made for the same amount it costs to finance a nicotine addiction. For example, one advertisement estimated vaping costs about $830 per year if the individual vapes the nicotine equivalent of 4 packs of cigarettes per week. In this example alternative purchases should be things like concert/athletic event tickets, cruise tickets, or monthly rent payments as opposed to student loan debt [(3), Figure 2]. Overall, the financial impact of nicotine addiction serves as a particularly powerful deterrent, especially among college students who often lack large financial resources (8).

### Figures of speech

Use of metaphors and similes when clear, accurate, and familiar are considered an effective means of communication. However, when exaggerated, confusing, or unfamiliar they may do more harm than good. For instance, one anti-vaping advertisement described parasites eating away at the brain. A similar campaign showed parasites crawling through the body causing severe deformities. In reality, no parasites are actually transmitted to the body through vaping; this is just a metaphor for addiction. However, some viewers may not know this taking the PSA literally. If and when the viewer finds out that what was said in the advertisement was not actually true, this will hurt the credibility of the organization that produced the advertisement. In turn, teenagers who view the warning may discount anything the creators of the advertisement claim in the future. Thus, metaphors which make exaggerated claims should be strictly avoided as they are unlikely to elicit the hoped for response (6).

Similes in PSAs may also fail to accurately depict nicotine addiction. For example, in one campaign to represent the permanency of nicotine addiction they compare it to a face tattoo [(7), Table 2]. The issue with this is that it implies that an individual who is addicted to nicotine will be a lifelong user, and that their addiction is outwardly visible. Not only is the latter false, but it does not encourage cessation. A slightly altered version of this PSA, comparing nicotine addiction to laser tattoo removal is better as this would demonstrate that it is possible to quit using nicotine but it is also painful and difficult which is why one should avoid using products that contain it. In essence, the use of similes and metaphors, when implemented correctly can be an effective means of translating information to teens about the dangers of vaping. At the same time, they should be used with caution as these figures of speech can often be misinterpreted by teens in a way that may spread misinformation and discourage cessation. Put simply, scenarios depicting vaping consequences should be realistic and believable to youth; not overexaggerated (22).

### Teenage vernacular

Young people do not like to be mocked or talked down to. Thus, anti-vaping messages simulating adolescent or young adult trends by using teenage vernacular or perspective (2) are not popular. This includes the use of memes and hashtags as well as first and third person language such as “I” and “we” and the word “teen.” Both of these significantly lower perceived message effectiveness. Preferable messages use second person language such as “you” which directly engages the viewer without trying to mimic them [(12), Table 5]. In addition to this, messages considered to be accusatory, shaming, threatening, aggressive, or authoritative are also viewed as counterproductive (8). An example of this is reminding students of tobacco free campus policies. This gives off a parental tone that was rejected by those who viewed this message (3).

### Product depiction

Research clearly shows that bright colors, mention of flavors (12) and showing people vaping (30) decreases risk perception, increasing vaping appeal which is why these elements are often used in vaping advertisements. Thus, anti-vaping PSAs should avoid these features. Instead, the goal of an anti-vaping PSA should be to look like the opposite of their advertisement counterparts by associating vaping with negative rather than positive imagery (12). For example, darker hues like black and white could be used to evoke a sense of death and dying (30). Finally, the product itself should be illustrated to avoid confusion. However, if a person is shown with the product, they should only be depicted holding it (22) not using it as the latter may glamorize vaping and encourage use (11).

### Environmental impact

Vaping litter is not biodegradable and as such is considered harmful to the environment (12). Disposable vape pens which have recently resurged in popularity have made this even worse. Unlike traditional cigarettes which are made of paper, foam and tobacco leaves, e-cigs are mostly made of plastic and contain e-liquid which is toxic to animals if consumed. Regardless, this is considered the least impactful message theme, ranking lower than nicotine addiction (31) implying that it should be removed from any anti-vaping messages as it is unlikely to convince young people not to vape.

### Vaping industry

Today, all major tobacco companies have at least one or more e-cigarette products in their portfolio. JUUL is perhaps the most well-known example of this, after 35% of the company was acquired by Altria, the manufacturer of Marlboro cigarettes in 2018. This decision by tobacco companies is part of a strategy to expand their customer base [(6), p. 4] that has been slowly dwindling as cigarette use has become significantly less popular, especially among teens and young adults. It is therefore not surprising vape companies have used the same marketing techniques as tobacco companies once did, targeting schools and summer camps to promote their products (7). Furthermore, to gear their products more toward youth, they have engineered vapes to look strikingly different from their combustible counterparts. This has been done on purpose since research indicates that things associated with cigarettes such as tobacco flavor, are rated as more harmful and are therefore less likely to be used by teens (29). In addition to this most leading e-cigarette companies market their products specifically to children and those under the age of 21, by not using age restriction tools. As a result, about 40% of youth [(4), p. S19] have been exposed to digital tobacco marketing, increasing their chances of using these products.

Yet, despite being told the above in anti-vape messages most teens report being unmoved by deceptive industry messages. The primary reason for this is that Generation Z has the lowest levels of trust in corporations compared to any other generation. Therefore, the vast majority believe that businesses have no ambition beyond profit in turn making the manipulative tactics of vape companies expected and unsurprising. Other research claims that anti-vaping campaigns that focus on the tobacco industry fail because teens are not vaping to rebel, but rather to fit in, socialize and engage with new flavors and technology (7). The last reason provided for why these sorts of messages might fail is based on the implementation of the message. For example, one study found that messages that told teens they were the tobacco industry's dummy or guinea pig, were viewed as “mean” [(11), Figure 2]. However, some teens disagreed with the aforementioned mentality. Instead of feeling personally attacked by the messages, they found them reassuring as they put the blame on society/the industry rather than the individuals or vapers themselves (16). In conclusion, messages discussing vape industry ploys provide mixed results but cannot be definitively ruled out as ineffective.

### Mental health

Similar to themes regarding industry deception, there is also little consensus as to the true impact vaping has on mental health. This is a common theme in both *Truth and The Real Cost* campaigns which claim that vaping increases depression and anxiety (4, 7). Three of the studies analyzed explored this theme. One study determined that because mental health effects are more short term, participants were more likely to care about them. Thus, if vaping use really was associated with increased rates of anxiety and depression, this would be a significant motivator to not vape. The issue is that some questioned whether vaping caused mental health issues or if people were choosing to vape to cope with pre-existing conditions (7).

Another study focusing on college students suggests the latter, relating that vaping is often used as a mental health management tool (3). Considering nicotine is a stimulant it would make sense that some would use it as a cheap way to combat depression. Regarding anxiety, the connection is less clearcut as anxiety is usually a withdrawal symptom of nicotine reduction. Thus, nicotine would not relieve anxiety symptoms unless the individual was already addicted to the substance. This may explain why college students vape more during stressful time periods (3). However, among those not addicted to nicotine, anxiety is unlikely to be reduced by vaping. This is supported by a third study which implied that initiating nicotine use can cause anxiety in people who had not previously exhibited these symptoms as non-users (4). In conclusion, the link between mental health problems and vaping is limited and unclear demonstrating the need for message pre-testing before claims like these are disseminated to the public.

## Discussion

### Implications

#### For literature

This review significantly improves upon previous literature on the topic by adding the specificity needed to make actionable changes to current anti-vaping campaigns and messages. First, this paper examines message themes other than health harms and addiction. This is something that the meta-analyses on anti-vaping campaigns (24) failed to do, including only one study that assessed the influence of marketing strategies of tobacco companies on message effectiveness. In turn, this made it impossible to assess if this factor and numerous others had any effect on vaping behavior.

This essay also examined studies that assessed the impact of emotionally provocative messages, meaning that the messages contained images as opposed to only text. This was an important factor to investigate since disturbing visuals are more likely to change risk beliefs than text alone (23). Perhaps most importantly this evaluation revealed that nicotine addiction messages, though one of the most popular themes in vaping prevention messages, are one of the least effective, directly contradicting previous claims made by Ma and colleagues (24). In essence the previous meta-analysis (24) on the subject lacked the heterogeneity needed to fully assess anti-vaping messages. Thus, by assessing a larger variety of message effects and different aspects of anti-vaping prevention messages this assessment adds to the current literature by providing a significantly more comprehensive analysis of different message themes.

#### For policymakers

The primary implication of the above results is the need to expand upon the current FDA warning label on all vapor product packaging. At the moment, the warning simply asserts that nicotine is addictive. It does not emphasize the consequences of nicotine addiction; health related or otherwise (23). It also does not mention any of the toxins or other chemicals found in vapor products and the numerous health problems they have been linked to including cardiovascular disease, brain diseases, altered memory capacity, increased probability of cancer, and pulmonary illness (5). Therefore, the FDA warning label should be significantly altered to contain specific health implications of vape and nicotine use.

For reference, the FDA could use the label on the original MarkTen e-cigarettes. This label goes far beyond the FDA warning that nicotine is addictive, noting that nicotine increases the heart rate and blood pressure and that e-cig vapor may aggravate respiratory harm. Furthermore, it states that the product is not recommended for use by children, pregnant or breastfeeding women, individuals with heart disease or high blood pressure, or those with asthma [(28), Figure 1]. Thus, the MarkTen label encompasses numerous health harms associated with vaping and nicotine use. However, it lacks brevity; thus, similarly to the current labels on cigarette packages, the FDA should separate the MarkTen warning label into multiple messages, alternating them to include different ones on different packages (32). The FDA could also add to these statements more recent health implications of vaping such as the toxins found in e-cigarette vapor. Alternatively, the label could simply state “medical authorities recommend individuals refrain from the recreational use of this product as this practice may result in serious lung damage [(1), p. 95].” Either way changes like these would significantly improve the FDA's current health warning on e-cigarette packages.

Furthermore, though not the focus of this paper, health communication campaigns must also be accompanied by structural changes that would restrict youth and young adult access to e-cigarettes. Popular recommendations supported by college students include a price increase (3), restricting price discounting and other promotions (2), enforcement of clean indoor air laws particularly in bars, increasing the purchase age (3), limiting e-liquid flavors of disposable devices, and controlling retail licensing by removing the sale of devices from easily accessible purchasing locations like gas stations and convenience stores (2, 3). Other considerations include limiting the nicotine content of vapes or requiring a prescription to obtain one (20). Also given the recent surge in use of disposable vape pens (14) the FDA could require these products be less colorful and emit less vapor to deter use by children who might find these elements particularly appealing.

### Limitations

In the studies used for this review, the most recent data was collected in 2021. While this may seem fairly recent, this is almost 5 years ago, and a lot has happened since then. For example, a tiktok video went viral in 2022 depicting the insides of a popular disposable vape pen. The video revealed that the white cotton inside the device, was a dark yellow/orange closely resembling the filter of a cigarette after it has been smoked (33)^1^ Other videos depicted individuals' agonizing hospitalization experiences due to heavy vaping or their difficulty trying to quit vaping (34)^2^ When combined, all of these factors could have a significant impact on one's perception of vaping risk. Therefore, it is possible that some individuals who previously reported being unphased by the supposed health effects of vaping (30), would be more likely to believe the claims now. Another limitation of this analysis is that it only includes 16 studies, which is mostly due to the fact anti-vaping campaigns are fairly new and thus studies examining their effectiveness are few in number.

Beyond that, this appraisal is only generalizable to the adolescents and young adults living in the US. Various approaches have been taken in different countries to combat the vaping epidemic. For instance, in Australia a prescription is required to buy nicotine vapor products (20). In the US, you do not need this, nor do you have to prove that you were previously a smoker or addicted to nicotine. Furthermore, as much as 50 or 60 mg/ml of nicotine can be found in a single vape sold in the US. That is 2.5–3 times as much nicotine as a vape available for purchase in the UK, where the nicotine content of vapes is limited to 20 mg/ml (20, p.6). The impact of these two facts on the US population is that young people may be more likely to vape due to the fact it is easier to purchase them and they are more addictive. In turn these differences, may alter one's views on the dangers of e-cigarettes. This demonstrates how the effectiveness of specific vaping prevention messages may vary depending upon the exact population they are trying to target. Lastly, a final limitation is that some individuals might have been influenced by additional material they viewed outside of the anti-vaping messages reviewed in the studies compounding their views and the results of the review.

### Future research

In general, future researchers should conduct message pretesting before a campaign is released to the public (4). This entails fact checking scientific claims as well as eliciting advice from the target audience of the campaigns. Both are essential to ensuring resources are not wasted, and that the organization responsible for producing the ad does not lose its credibility by making unsubstantiated claims or alienating their audience (12, 22). The latter is of particular importance with regard to this topic, since what messages adult researchers think will be effective or relatable to kids may not actually be (11). Engaging with the target audience also ensures that messages are not just targeting the correct audience but also the correct product. For example, the use of nicotine pouches like Zyn, has increased in recent years, particularly among young adult males [(15), Table/Figure 96]. With that being said, the overall percentages of young adults and teens who use nicotine pouches is very low when compared to the percentage of teens and young adults who vape [(35), p. 33]. This relates to campus culture, which youth are often more aware of than their adult counterparts conducting the research (3). This demonstrates how pretesting will increase both the authenticity and effectiveness of anti-vaping campaigns.

Future research may also want to look into whether stated intentions not to vape actually correlate with subsequent behavior and use. This is known as the *attitude-tendency inconsistency* and was observed in the context of vape consumption in the meta-analysis noted in the literature review on vaping advertisements (5). Furthermore, in the meta-analysis on vaping messages, Ma and colleagues (24) found that though vaping prevention messages decreased vaping intentions, the effect was small when compared to the impact on risk beliefs. This indicates that behavior may be more difficult to change than the risk beliefs themselves (24). Noar and colleagues (23) also noted this problem particularly with regard to nicotine addiction, which is often discounted by youth. Put simply, just because somebody knows something is risky doesn't necessarily mean they aren't going to do it. This is especially true when the consequences of those behaviors are far in the future. With that being said, the numerous cases of young people that have been hospitalized as a result of heavy vaping might help to change this sentiment (34). Additionally, changes in beliefs are generally considered to be valid precursors to changes in behavior according to the Theory of Planned Behavior (4). Regardless, this would be an interesting topic for a future paper, focusing only on longitudinal studies which examine behavior of individuals over time (22). This would also make the results of analysis more accurate as they would be based on observed behavior rather than predicted behavior.

## Conclusion

In conclusion, despite the widespread notion that anti-vaping campaigns do not discourage vaping initiation or encourage vaping cessation research shows that depending on the message some can. Specifically, messages that focus on the known short term health effects of vaping and those that mention the chemicals found in vapes and their harmful effects are most effective. The use of graphics further assists in this regard though health comparison statements, bright colors, mention of flavors and images of people vaping does not. Testimonials from influencers who are former vapers shared via social media also show promise. Meanwhile messages that focus on the addictive nature of nicotine without alluding to the health impacts and financial costs of addiction are the least effective along with references to vaping litter which rank the lowest. The use of teenage vernacular and authoritative tones are also ineffective. With regard to policy implications, there is an obvious need for a more formidable FDA warning on vapor product packaging in order to have a significant impact on vaping initiation and cessation. In summary, campaigns must be improved to prevent a surge of vaping related illness and potential death as well as the renormalization of smoking and nicotine addiction among youth and young adults.

## Footnotes

1
https://nypost.com/2022/09/06/tiktoker-exposes-disgusting-insides-of-vaping-devices/

2
https://nypost.com/2022/07/07/my-lung-spontaneously-collapsed-vaping-i-could-have-died/
