# Supplementary material for: The effectiveness of anti-vaping health communication campaigns among high school and college students in the U.S
Source: Front Public Health. 2026 Jan 5;13:1676181. doi: 10.3389/fpubh.2025.1676181 (PMC12850514; doi:10.3389/fpubh.2025.1676181)
Supplement: Supplementary file 1 [file Table_1.docx]

**Supplementary Table 1:** Message Themes That Influence Vaping Cessation

| **Article Title** | **Methods** | **Findings** |
| --- | --- | --- |
| Perceived Effectiveness of Objective Elements of Vaping Prevention Messages Among Adolescents (12) | In 2020, a national sample of 1,501 US adolescents (13-17, 30% vapers, mostly white) completed an online survey in which they were asked to rate 7 different vaping prevention messages from a pool of 220 on Perceived Message Effectiveness (PME) and vaping appeal. | - Message themes about nicotine addiction, chemicals, health effects including death and comparison to cigarettes, were associated with greater PME. - Industry targeting, flavors and environmental impact were associated with lower PME, with flavors increasing vaping appeal. - Imagery associated with higher PME included warning symbols, graphic images, and combustible cigarettes. - Use of hashtags, statistics, bright vivid colors, memes and the use of first person or the word “teen” were all associated with lower levels of PME. |
| Rethink Vape: Development and Evaluation of a Risk Communication Campaign to Prevent Youth E-Cigarette Use (11) | Between 2015 and 2016 Southeastern Virginia teens ages 11-19 were surveyed (674) and participated in focus groups (82) assigned to evaluate the *RethinkVape* campaign. | - Vapor content, health risk such as lung disease and big tobacco marketing ploys should be the focus of anti-vaping campaigns. - Wordplay and metaphors should be skipped as they may be misunderstood by teens or viewed as “mean”. - To avoid normalizing teen use, any model shown in an ad should be seen holding the product not using it. - When discussing health effects, avoid talking about “how little” we know. |
| Developing a Targeted E-cigarette Health Communication Campaign for College Students (3) | In 2019, a convenience sample of 98 students from a Midwestern University were separated into 7 focus groups to provide feedback on sample anti-vaping messaging. | - Statistics indicating only a minority of college students vape do not seem to be effective for vape users. - Possibility of addiction and financial cost seen as barriers to use. - Messages should refrain from using terms like “addiction” or “shame”. - Uncertainly about the long-term health effects of e-cigs, is often a justification for use, and should be avoided in anti-vaping messages - Messages with information on the chemicals contained in e-cigs seem to be the most effective, since they cause individuals to feel a sense of fear. |
| Youth Perceptions of E-Cigarette-Related Risk of Lung Issues and Association With E-Cigarette Use (26) | In 2020, a national cross sectional convenience sample of 4,315 US adolescents and young adults ages 13 to 24 (50% non-users and users) took an online survey regarding their perception of lung issues associated with vaping. | - Users perceived a lower risk of respiratory problems and illness compared to non-users. - Past months use more likely among those who believe there is no hard evidence connecting vaping to severe lung disease. - Past month use of e-cigs was less likely among youth and those who believe vaping could cause respiratory harm. |
| Comparing belief in short-term versus long-term consequences of smoking and vaping as predictors of non-use in a 3-year nationally representative survey study of US youth (25) | Over a span of three years (2014-2017), a nationally representative group of adolescents and young adults between 13 and 26 were surveyed via phone on the consequences of using both combustible and noncombustible tobacco products. 4,470 of them were asked about e-cigs. | - Short term consequences more likely to lead to anti vaping intentions and subsequent behavior - This is due to temporal discounting by adolescents and young adults - Friend disapproval was most strongly associated with intention not to use tobacco products. |
| High School Youth and E-Cigarettes: The Influence of Modified Risk Statements (MRSs) and Flavors on E-Cigarette Packaging (28) | During the 2016/17 academic year, 657 high school aged youth (grades 9-11, ages 14-18, 18.4% non-smoking e-cig users) from 4 different high schools near a city participated in an experiment testing perceptions of and intentions towards use of e-cigs after viewing 3 kinds of e-cig warning labels (abstract, MRS, FDA) on 3 different brands of e-cig packaging (blu, MarkTen, Vuse). | - The greater perceived risk the lower the intention to use, even if the person has used in the past. - MarkTen label had higher risk perception than an abstract warning label (long term health risks are unknown) - MarkTen label generated higher message comprehension when it did not include an MRS (healthier than cigs) - MarkTen labels increased both ambiguity and counterarguing - The presence of conflicting information on the package leads to more active engagement with the arguments in the message. |
| Message and Delivery Preferences for Online Tobacco Education among Adolescents and Young Adults (8) | In 2016, 4 focus groups of 39 adolescents and young adults (16-26) from North Carolina consisting of mostly White users and susceptible non-users were asked to evaluate 3 pre-existing tobacco education websites (Real Cost, Truth, Still Blowing Smoke) | - Preference for new, concise facts with sources. - Aversion to scare tactics, attempts to follow youth trends (like hashtags), or authoritative tones. - Use of social media increases engagement - Messages should avoid blaming users and should instead place blame on the tobacco industry. |
| Social Media Message Designs to Educate Adolescents About E-Cigarettes (29) | In 2019, a national convenience sample of 928 highschoolers (15-18, mostly White) were recruited for an online experiment to test the effectiveness of message content in social media anti-vaping campaigns. | - Social media messages led to greater knowledge and beliefs about the harms of e-cigs. - Topics like lung damage, uncontrolled moods and ingesting specific harmful chemicals elicited higher intended message reactions. - Conveying the negative effects of nicotine exposure elicited muted message reactions. - Messages about burns from E cigarettes exploding and the sleek product designs of E cigarettes were rated the lowest for intended message reactions. - Negative effects on one’s social circumstances, (acceptance and popularity) health or appearance are most likely to change risk beliefs. |
| Optimizing Messages and Images for E-Cigarette Warnings (16) | In 2021, a national convenience sample of 1,629 US adults most of whom were White and either vaped (15%), smoked (43%) or did both (40%) took an online survey in which they evaluated three kinds of e-cigarette warnings (text, FDA required warning, images). | - New warning themes all discouraged vaping more than the current FDA warning - The most discouraging warnings were about toxic metal exposure. - Images of harms discouraged vaping more than images of hazards. - Images of internal harms or people experiencing harms were the most promising pictorial messages. |
| “Don’t do vape, bro!” A Qualitative Study of Youth and Parent’s Reactions to E-Cigarette Prevention Advertisements (6) | In 2019, 12 focus groups of 63 youth (11-18, 11% vapers) and 27 parents (22-77, mostly Black, 26% vapers) living in the Southeastern United States were surveyed and questioned regarding e-cigarette prevention messages. | - Chemicals in e-cigs perceived as new and scary; unknown chemical names caused confusion. - Youth most impacted by images visualizing the health effects of vaping and nicotine’s effect on behavior. - Participants learned that Big Vape=Big Tobacco - Some did not believe the financial costs of vaping were significant. - Participants recommended featuring testimonials and targeting youth already addicted to e-cigs. - Increasing diversity was also recommended. |
| Identifying Promising Themes for Adolescent Vaping Warnings: A National Experiment (31) | In 2020, a national probability sample of 623 adolescents (ages 13-17, majority White & 20% Hispanic, 14% current vapers) were surveyed regarding the impact of various warning message themes (litter, nicotine addiction, chemical harms, lung harms, COVID harms) | - Chemical, lung, and COVID-19 warning messages rated higher on PME and negative affect than nicotine addiction and control. - Nicotine addiction warnings were more effective than themes about vaping liter. - Causal language recommended to enhance understanding of the warning text. |
| Creating More Effective Vape Education Campaigns: Qualitative Feedback from Teens in Nine U.S. States (7) | A total of 337 teens from 9 US states (Hawaii, Indiana, Kansas, Kentucky, Nevada, Oklahoma, South Carolina, Vermont, Virginia) aged 13 to 18 who vaped or were susceptible nonusers participated in in-person and online surveys, focus groups and interviews between the years 2018 and 2021 to review the *Behind the Haze* vaping education campaign. | - Effective commercials included detailed facts with explanatory visuals, metaphors, and empathy. - Promising topics include chemicals, physical consequences and mental health, and the impact of vaping on others. - Addiction and industry deception messages were less impactful. - Empathy preferred over judgment, fear mongering and lecturing. |
| Identifying Message Content to Reduce Vaping: Results from Online Message Testing Trials and Young Adult Tobacco Users (30) | In 2020, a convenience sample made up of 969 Amazon Mechanical Turk workers (most of whom smoked/vaped) ages 18 to 24, living in the US, who use 1 or more social media sites weekly were surveyed regarding educational e-cig messages/images. | - Flavor and social message themes decreased PME of harm messages and should be avoided. - Messages focused on the harms of vaping produced the highest PME scores. - Addiction messages are least discouraging to adult tobacco users. |
| The Impact of E-Cigarette Warnings, Warning Themes and Inclusion of Relative Harm Statements on Young Adults E-Cigarette Perceptions and Use Intentions (27) | In 2018, an online experiment was conducted on 876 young adults (mostly white, ages 18-29, living in the US, 6.7% nonsmoking e-cig users) recruited through Amazon’s Mechanical Turk testing the impact of different warning messages in relation to theme (nicotine addiction, nicotine’s impact on the brain, presence of harmful chemicals) and type (relative harm vs standard warning). | - PME was higher for the brain and chemicals warnings compared to the addiction warning. - Warnings with the relative harm statement (e-cigs may cause harm to health but are less harmful than cigarettes) were less believable and credible and less likely to deter e-cig use. - Relative harm warning increased the use of E cigarettes for harm reduction purposes by smokers. - Inclusion of relative harm statements seemed to reduce recall of the risk related part of the message. |
| Impact of Vaping Prevention Advertisements on US Adolescents A Randomized Clinical Trial (23) | In 2021, 1,514 adolescents were recruited online (13-17 years old, susceptible to vaping or current vapers/tobacco users, mostly White, ¼ Black) to take part in a randomized clinical trial in which they were surveyed about the impact of the *The Real Cost* vaping prevention campaign. Participants were separated into three groups each testing a different message theme (health harms, addiction and control) | - Those who viewed *The Real Cost* (health harms and addiction) advertisements had lower vaping and smoking susceptibly than those who viewed the control group video (text on screen talking about product definitions, farming and manufacturing practices) - Health harms advertisements discussing toxic substances and lung damage performed marginally better than addiction advertisements. - Video advertisements like *The Real Cost* expand upon the consequences of nicotine addiction compared with current vapor product packaging which does not. |
| Evaluating *The Real Cost* Digital and Social Media Campaign: Longitudinal Effects of Campaign Exposure on E-Cigarette Beliefs (4) | Between 2020 and 2021, a nationally representative longitudinal cohort of 2,625 youth (11-16 year olds) were surveyed about 11 vaping related beliefs after viewing 6 *Real Cost* vaping advertisements. | - Use of digital advertising to disseminate *The Real Cost* ads was highly effective as 75% of youth had seen at least one add previously. - Social media use decreases agreement with anti-vaping beliefs. - Exposure to ads was associated with increases in beliefs with themes discussing health harms/toxic chemicals, anxiety, future cigarette use, and “disappointing people important to youth”. - No evidence was found that exposure changed beliefs regarding addiction or “disappointing family” themes. |
