# Supplementary material for: The effectiveness of anti-vaping health communication campaigns among high school and college students in the U.S
Source: Front Public Health. 2026 Jan 5;13:1676181. doi: 10.3389/fpubh.2025.1676181 (PMC12850514; doi:10.3389/fpubh.2025.1676181)

Supplementary Figure 1. PRISMA flow diagram demonstrating the selection process and the number of studies identified, screened, excluded and included at each stage.

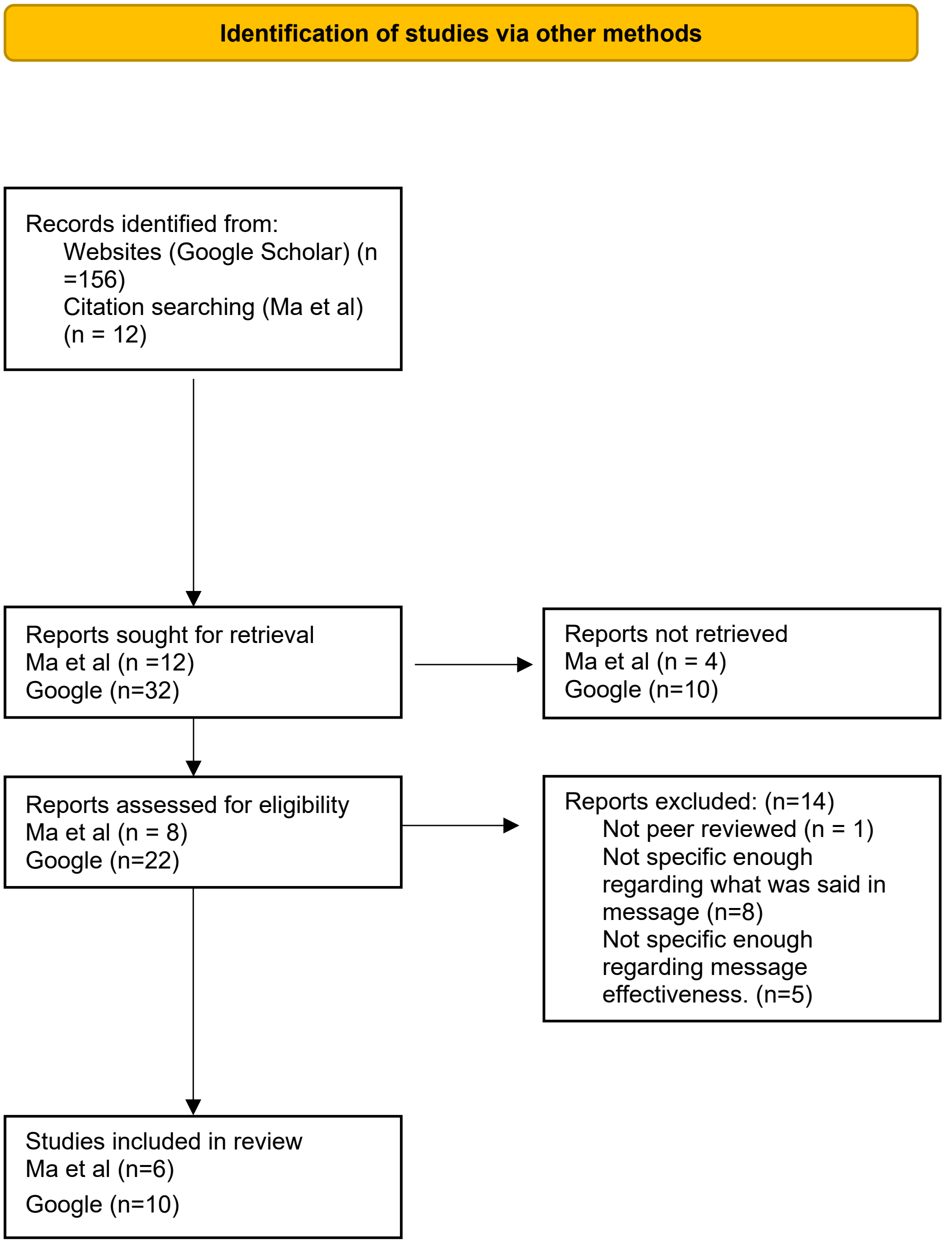

Supplement: Supplementary file 2 [file Image_1.pdf]
